# Fracture Risk After Corticosteroid Injections in Non-geriatric Patients With Upper Extremity Osteoarthritis: A Retrospective Analysis

**DOI:** 10.7759/cureus.97766

**Published:** 2025-11-25

**Authors:** Edward L Major, James R Goetz, Delaney Shroat, Christopher J Fleisher, Chaitu Malempati

**Affiliations:** 1 Medicine, University of Kentucky College of Medicine, Lexington, USA; 2 Orthopaedic Surgery and Sports Medicine, University of Kentucky College of Medicine, Lexington, USA

**Keywords:** bone health, corticosteroid injection, fracture risk, non-geriatric, osteoarthritis, upper extremity

## Abstract

Background: Corticosteroid injections (CSIs) are commonly used for symptomatic management of upper extremity osteoarthritis (OA). While systemic corticosteroids are associated with impaired bone health, the fracture risk of intra-articular CSIs in younger patients remains unclear. This study aimed to determine whether intra-articular CSIs of the shoulder, elbow, or wrist increase short-term fragility fracture risk in non-geriatric patients with upper extremity OA.

Methods: We retrospectively reviewed patients aged < 65 years who received intra-articular CSIs in the shoulder, elbow, or wrist between 2018 and 2023. Patients with prior upper extremity fractures, ipsilateral shoulder arthroplasty, metabolic bone disease, or medications affecting bone metabolism were excluded. Data on demographics, injection history, and fracture occurrence were collected and analyzed using chi-squared tests and logistic regression, with significance set at p < 0.05.

Results: Among 431 patients (mean age = 45.5 ± 10.64 years; 46.4% female), the fracture incidence after CSI was 1.9% (8/431) over a mean follow-up of 390 days. Fracture risk was not significantly associated with age (p = 0.92), sex (p = 0.36), body mass index (p = 0.11), smoking status (p = 0.24), injection site (p = 0.99), or injection frequency (p = 0.533). Logistic regression confirmed no association between the number of injections and fracture occurrence (p = 0.61).

Conclusions: Intra-articular CSIs of the shoulder, elbow, or wrist were not associated with an increased short-term fragility fracture risk in non-geriatric patients with upper extremity OA. These findings support the continued use of CSIs in younger populations, although long-term and higher-risk cohorts warrant further investigation for broader risk assessment.

## Introduction

Background

Osteoarthritis (OA) of the upper extremity, including the shoulder, elbow, and wrist, is increasingly diagnosed in younger, active individuals [1] and contributes significantly to clinical and economic burdens [2]. Intra-articular corticosteroid injections (CSIs) are frequently used for symptomatic management, providing pain relief and delaying surgical intervention [3,4]. Despite their widespread use, concerns remain regarding their long-term safety, particularly with respect to bone integrity and the risk of fragility fractures [5].

Systemic corticosteroids are well-documented to impair bone metabolism and increase the risk of fragility fractures [3]. Upper extremity fragility fractures, such as distal radius or proximal humerus fractures, have been shown to increase the risk of subsequent hip fractures by up to fivefold within one year [5]. Meta-analyses confirm that both current and prior corticosteroid use independently increase fracture risk, regardless of bone mineral density or prior fracture history [6,7]. While intra-articular CSIs are designed to deliver localized effects, systemic absorption may not be negligible and has raised safety concerns, especially in older adults and individuals with compromised bone health [8].

Most studies evaluating fracture risk in the context of corticosteroid use have focused on weight-bearing joints and older or osteoporotic populations [8-10]. Recent literature highlights the need for more comprehensive studies to better understand the long-term skeletal effects of CSIs, particularly in young adults [4,8,11]. However, real-world data specific to upper extremity CSIs in non-geriatric populations remain limited, making it difficult to offer evidence-based guidance for clinicians treating younger patients with upper extremity OA.

Objective

This study aimed to determine whether intra-articular CSIs administered to the shoulder, elbow, or wrist are associated with an increased short-term (~one year) risk of upper extremity fragility fractures in either upper extremity, regardless of laterality, in non-geriatric patients with upper extremity OA. We hypothesized that CSIs would not be associated with an elevated risk of fragility fractures in this younger population.

## Materials and methods

Study design and setting

This investigation was designed as a retrospective cohort study utilizing electronic health record (EHR) data from a single academic medical center. Patient identification and data extraction were performed using structured queries of the Meditech EHR system (Meditech, Canton, MA). The study cohort included all consecutive patients who underwent at least one intra-articular CSI in the shoulder, elbow, or wrist for the treatment of osteoarthritis between January 2018 and June 2023 at our institution.

Ethical approval

The study protocol was approved by the Medical Center Institutional Review Board (IRB #: 24-01-31-Goet-Fx-Up-Ext; approved: December 17, 2024) and was conducted in accordance with the Declaration of Helsinki and its amendments [12]. The requirement for informed consent was waived due to the retrospective study design and use of fully de-identified data.

Selection criteria

A total of 688 patients were identified and screened for inclusion in the study. Patients were eligible if they were aged < 65 years, had a clinical diagnosis of OA in the injected joint, and received at least one intra-articular CSI in the shoulder, elbow, or wrist joints. To reduce confounding factors known to affect bone integrity, the following exclusion criteria were applied: (1) history of ipsilateral upper extremity fracture; (2) diagnosis of osteoporosis, osteopenia, or metabolic bone diseases (e.g., osteogenesis imperfecta, Ehlers-Danlos syndrome, and muscular dystrophy); (3) history of ipsilateral shoulder arthroplasty; (4) use of medications known to alter bone metabolism (e.g., bisphosphonates, thyroid hormone, and antiepileptics); (5) non-intra-articular (e.g., bursa and intramuscular) or non-upper extremity injections (e.g., knee and hip); and (6) non-steroidal injections (e.g., viscosupplementation). The full exclusion and selection criteria are presented in Table 1.

**Table 1:** Patient exclusion and selection criteria.

Exclusion criteria		n
Non-upper extremity injection		150
Osteoporosis/osteopenia		14
Previous upper extremity fracture		10
Metabolic bone disease		
	Osteogenesis imperfecta	1
	Ehlers-Danlos	2
	Muscular dystrophy	1
Non-steroidal injection		
	Viscosupplementation injection	6
Non-intraarticular injection		
	Bursal injection (not including the subacromial bursa)	5
	Intramuscular injection	1
At-risk medication use		
	Thyroid replacement therapy (levothyroxine)	57
	Antiepileptics (phenytoin, carbamazepine)	2
Other		
	Ipsilateral shoulder arthroplasty	4
	Deceased (no follow-up)	2
	Duplicate patient	4
	No injection	2
Total patients excluded		261

Data collection

Three independent reviewers (E.L.M., J.R.G., and D.S.) retrospectively collected data from the medical records of patients who met the inclusion criteria. Data were collected and managed using Research Electronic Data Capture (REDCap, Vanderbilt University, Nashville, TN), a secure web-based data software [13,14]. Extracted demographic variables included age (years), sex (male or female), body mass index (BMI; kg/m^2^), and smoking status (current, former, or never user). Injection history was recorded, including the joint injected, the number of injections, and the dates of the first and last injections. Corticosteroid formulation and dose (e.g., triamcinolone acetonide and methylprednisolone acetate) were recorded in the EHR and available in the dataset; however, formulation type was not included as a primary study variable because the investigation was not designed to assess dose-dependent or formulation-specific differences in fracture risk. Triamcinolone acetonide was the most commonly used corticosteroid during the study period, consistent with institutional practice.

Fractures were identified during review of EHR documentation of all subsequent visits from the initial injection to the final follow-up. All interim clinical encounters within this period were reviewed to detect any new upper extremity injuries or suspected fracture events. When a potential fracture was identified, all available radiographs, CT imaging, and radiology reports were examined to confirm the diagnosis and ensure the injury met criteria for a low-energy fragility fracture. The AO/OTA (Arbeitsgemeinschaft für Osteosynthesefragen/Orthopaedic Trauma Association) classification was initially performed by study personnel and subsequently reviewed and verified by the supervising attending orthopedic surgeon. The attending physician's classification was used as the final determination for analysis.

For patients who sustained an upper extremity fracture after CSI, the fracture date, location, classification using AO/OTA standards [15], and mechanism of injury (classified as low energy, high energy, or unknown) were documented. Only low-energy mechanisms consistent with fragility fractures were included in the analysis. Fracture laterality (ipsilateral or contralateral to the injected extremity) was recorded for all fracture events based on imaging review and provider documentation. Fractures occurring in either upper extremity following CSI were included, as the aim was to evaluate overall upper extremity fragility risk rather than localized mechanical effects of injection. The duration from the initial injection to the final clinical follow-up was also recorded.

Statistical analysis

Descriptive statistics were reported as means with standard deviations (SD) for continuous variables and frequencies with percentages for categorical variables. Chi-squared tests were used to evaluate the associations between fracture occurrence and categorical predictors (age, sex, BMI category, smoking status, injection site, and injection frequency). Patients were categorized based on the total number of intra-articular injections received during the study period, with those receiving nine or more injections grouped into a “9+ injections” category to account for outliers. Logistic regression was used to assess the relationship between injection frequency and fracture risk, and Pearson’s correlation analysis was used to evaluate the linear relationship between the number of injections and fracture occurrence. The assumptions for these statistical tests, including the independence of observations, were verified. As this was a retrospective review, a formal a priori sample size calculation was not performed in this study. Instead, all eligible patients treated between January 2018 and June 2023 were included to capture the complete dataset for analysis. Analyses were performed using IBM SPSS Statistics version 29.0 (IBM Corp., Armonk, NY) and JASP (version 0.19.2). Statistical significance was set at p < 0.05 for all analyses.

## Results

Demographic characteristics

A total of 431 patients met the inclusion criteria. The mean age of the study population was 45.5 years (standard deviation (SD) = 10.6), and 46.4% (200/431) were female. The mean BMI was 30.7 kg/m^2^ (SD = 6.5). Smoking status included 31.1% current smokers (134/431), 17.6% former smokers (76/431), and 49.4% never smokers (213/431). The mean follow-up period was 389.8 days (SD = 558.1). Baseline demographic and clinical characteristics are summarized in Table 2.

**Table 2:** Study population demographics. Data reported as mean (standard deviation or percentage of total). BMI, body mass index; SD, standard deviation.

Characteristic	Total patients (n = 431)
Age, years (SD)	45.49 (10.64)
Female, n (%)	200 (46.4%)
BMI, kg/m^2^ (SD)	30.73 (6.5)
Smoking status, n (%)	
Current	134 (31.1%)
Former	76 (17.6%)
Never	213 (49.4%)
Total injections (SD)	1.9 (2.4)
Fracture present, n (%)	8 (1.9%)
Follow-up, days (SD)	389.80 (558.08)

Injection frequency and fracture occurrence

The mean number of upper extremity injections administered per patient during the study period was 1.9 (SD = 2.4). A total of eight patients (1.9%) experienced an upper extremity fragility fracture following CSI, with a mean interval of 914.4 days (SD = 426.0) between the last injection and fracture. Among the eight fractures identified, four (50%) occurred in the ipsilateral extremity and four (50%) in the contralateral extremity in relation to the injected limb. Fractures were located in the distal radius (3/8, 37.5%), humerus (2/8, 25.0%), and metacarpal/phalanx (3/8, 37.5%). All fractures were classified as low-energy injuries consistent with fragility mechanisms (Table 3). Chi-squared analysis showed no significant association between fracture risk and age (p = 0.923), sex (p = 0.357), BMI (p = 0.106), smoking status (p = 0.241), or injection site (p = 0.991).

**Table 3:** Fracture location and AO/OTA fracture classification. AO/OTA: Arbeitsgemeinschaft für Osteosynthesefragen/Orthopaedic Trauma Association.

Fracture number	Fracture location	AO/OTA classification	Description
1	Radius, distal	2R3A2.1	Radius, distal end segment, extraarticular fracture
2	Radius, distal	2R3A2.2	Colles fracture with dorsal displacement
3	Radius, distal	2R3C2	Complete simple articular fracture, metaphyseal multifragmentary
4	Phalanx, distal	78.4.3.1A	Distal segment fracture
5	Phalanx, proximal	78.5.2.1B	Avulsion fracture, partial articular
6	Metacarpal, diaphysis	77.3.2C	Multifragmentary diaphyseal fracture
7	Humerus, surgical neck	11A2.1	Simple surgical neck fracture
8	Humerus, surgical neck	11B1.1	Extra-articular bifocal fracture with greater tuberosity involvement (3-part)

Fracture risk by injection count

**Figure 1:**
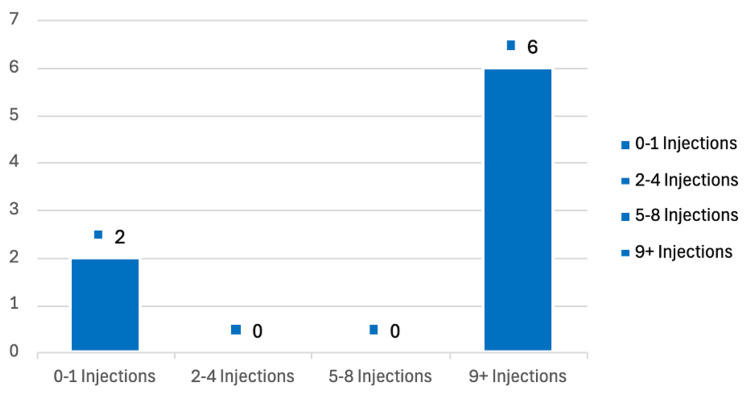
Number of fractures by injection count group.

When stratified by injection frequency, the fracture incidence did not increase with a higher number of injections. Figure 1 illustrates fracture distribution across injection frequency groups. Logistic regression confirmed that injection count was not a predictor of fracture risk (X² (1) = 0.390, p = 0.533). The total number of injections was not a significant predictor of fracture risk (β = -0.166, SE = 0.328, z = -0.508, p = 0.612).

Pearson’s correlation analysis revealed a weak negative correlation between the total number of injections and fracture occurrence (r = -0.023, p = 0.638). The 95% confidence interval for the correlation coefficient ranged from -0.117 to 0.072. The effect size measured using Fisher’s z-test was minimal (z = -0.023, SE = 0.048). Correlation analysis confirmed this with a non-significant Pearson’s r of -0.023 (p = 0.638).

## Discussion

Principal findings

In this retrospective analysis of non-geriatric patients with upper extremity OA, we found that intra-articular CSIs were not associated with an increased risk of fragility fractures over a mean follow-up of one year. The overall fracture incidence was low (1.9%), and no significant associations were observed between fracture occurrence and demographic variables, injection site, or injection frequency. These results suggest that CSIs remain a safe short-term treatment option for younger patients with upper extremity OA.

Comparison with existing literature

Our results align with those of prior studies reporting minimal fracture risk after intra-articular CSIs, particularly when used locally in patients without significant risk factors for bone loss [3,9]. While systemic corticosteroids are known to impair bone health and increase fracture risk [3], localized intra-articular formulations are designed to limit systemic exposure and are less likely to compromise skeletal integrity.

Sytsma et al. and Stadecker et al. similarly reported no direct correlation between localized CSIs and increased fracture incidence, though their populations were older and focused on weight-bearing joints [4,9]. Despite not observing an increased fracture risk with local CSIs, Sytsma et al. highlighted that the long-term effects and safety profiles of CSIs remain inconclusive [9]. Similarly, other studies have shown that older adults and individuals with pre-existing conditions that compromise bone integrity are at a higher risk of fracture after corticosteroid treatment [16]. Our findings contribute to the growing body of literature examining the safety profile of intra-articular CSIs, particularly as one of the only studies evaluating the upper extremity. Our findings in a healthier, younger sample underscore the importance of patient selection in evaluating CSI safety, as it continues to be used to treat the growing number of young OA patients.

Clinical relevance

This study helps fill a critical gap in the literature regarding the use of CSIs in younger patients with upper extremity OA. While OA is traditionally viewed as a degenerative disease of older adults, recent data suggest that more than half of OA cases occur in individuals under 65 years of age [1]. Given the high clinical utility of CSIs in delaying surgical intervention and improving function, establishing their safety profiles in this demographic is essential. Our findings provide reassurance that intra-articular CSIs do not appear to increase short-term fracture risk in younger patients without known bone disease. These results may support shared decision-making when considering injection-based management strategies for these patients.

Limitations

Despite the contributions of this study, several limitations warrant consideration when interpreting the findings. First, its retrospective design inherently limits the ability to establish causality and may have introduced selection bias. As with all retrospective EHR-based studies, our findings depend on the accuracy and completeness of clinical documentation, which introduces the potential for information bias. Second, the average follow-up period of approximately one year may not adequately reflect the delayed or cumulative systemic effects of CSIs on bone health. Sample size limitations, particularly the small number of fractures, may have reduced the statistical power to detect significant associations between the variables and precluded subgroup analyses. Although CSI formulations and doses were recorded, the present study did not analyze formulation- or dose-specific effects because this was not the primary aim. Given that systemic absorption may vary by corticosteroid type, future studies should evaluate whether specific formulations or cumulative steroid doses influence fracture risk. Because fracture events occurred in both the ipsilateral and contralateral extremities, the study may not isolate laterality-specific effects of CSIs on local bone integrity. Although not the primary objective of this study, the exclusion of older adults and those with comorbid bone conditions may reduce the generalizability of our findings to broader populations. Additional factors known to influence fracture risk, such as baseline bone mineral density, vitamin D status, dietary calcium intake, and physical activity level, were not routinely available in our dataset and may contribute to residual confounding.

Future directions

Prospective studies with larger cohorts, longer follow-up, and serial bone density assessments are needed to better characterize the long-term skeletal effects of CSIs. Future research should also explore potential interaction effects, such as whether smoking status, obesity, or cumulative steroid exposure modulates fracture risk, even in younger cohorts. Mechanistic studies examining systemic corticosteroid absorption from the upper extremity joints would further clarify the potential risk pathways.

## Conclusions

In this retrospective study of non-geriatric patients who received upper extremity CSIs for OA, short-term fragility fracture incidence was low, and no demographic or injection-related variable predicted fracture risk. These findings support the continued use of CSIs as a safe and effective component of nonoperative management in younger patients without underlying bone disease. Future prospective studies should evaluate long-term skeletal outcomes, cumulative dosing effects, and fracture risk in broader and higher-risk populations.
